# Effects of Qianlie Tongqiao Capsule on Bladder Weight and Growth Factors in Bladder Tissue of Rats with Testosterone-Induced Benign Prostatic Hyperplasia

**DOI:** 10.1155/2018/5059267

**Published:** 2018-11-05

**Authors:** Fan Zhao, Chun-he Zhang, Jun-feng Yan, Yan-feng Li, Yi-jian Yang, Di Sun

**Affiliations:** ^1^The Second Clinical Medical College, Zhejiang Chinese Medical University, Hangzhou 310053, China; ^2^Yunnan Provincial Hospital of Traditional Chinese Medicine, Kunming 650021, China; ^3^The First Affiliated Hospital of Yunnan University of Traditional Chinese Medicine, Kunming 650021, China; ^4^Zhejiang Hospital, Hangzhou 310013, China; ^5^Clinical Medical College, Yunnan University of Traditional Chinese Medicine, Kunming 650051, China

## Abstract

Qianlie Tongqiao Capsule (QTC) is clinically confirmed to be efficacious and safe in treating lower urinary tract syndromes and bladder dysfunction that are induced by benign prostatic hyperplasia (BPH). However, the functional mechanisms of QTC remain unclear. We aim to investigate the effects of QTC on both bladder weight and several growth factors in the bladder tissue of rats with testosterone-induced BPH. BPH in the rats was established through bilateral orchiectomy and subcutaneous administration of testosterone propionate (5 mg/kg) dissolved in corn oil. At the end of the study, all bladder tissues were collected and weighed, and a histological examination was conducted using H&E staining. Immunohistochemistry and quantitative reverse transcription-polymerase chain reaction (qRT-PCR) were applied to detect the expression of nerve growth factor (NGF), basic fibroblast growth factor (bFGF), and transformation growth factor-*β*1 (TGF-*β*1) in the bladder tissue. The expression of Bcl-2 and Bax in the bladder tissue was tested by Western Blot and qRT-PCR. We found that QTC, especially when administered in high-dosages, had a significant inhibitory effect on bladder weight gain and overexpression of NGF, bFGF, and TGF-*β*1 in rats with BPH. In addition, QTC downregulated and upregulated protein and mRNA expression of Bcl-2 and Bax in the bladder after prostatic obstruction, respectively. Furthermore, QTC balanced the Bcl-2/Bax ratio. Overall, these results reveal possible functional mechanisms of QTC in treating BPH-caused bladder dysfunction, and further studies are needed.

## 1. Introduction

Benign prostatic hyperplasia (BPH), which causes bladder outlet obstruction (BOO) and lower urinary tract symptoms (LUTs), is increasingly prevalent among aging males [1]. Nearly 50% of men aged >50 years will have pathological evidence of BPH, with the percentage increasing to >80% as men reach an age of >80 years [1, 2]. The pathophysiological mechanisms by which BPH leads to LUTs are still vague, but it is known that an enlarged prostate plays a role in BPH-induced BOO/LUTs. Nevertheless, even after undergoing obstruction relief surgery such as prostatectomy, many patients still report persistent storage symptoms [3–6]. Accordingly, many scholars have turned their attention to BPH/BOO-caused bladder damnification [7, 8]. Mirone V. et al. called the detrusor an “innocent victim” of BOO [7]. Mechanical obstruction originated from benign prostatic enlargement leads to a series of structural and functional changes in bladder detrusor [1, 7].

The bladder hypertrophy and detrusor smooth muscle cell hyperplasia that are caused by sustained pressure overload have been confirmed in both in vitro and in vivo studies [9–13], and both bladder hypertrophy and detrusor hyperplasia directly cause an increased bladder weight [7]. This change in bladder mass could be deemed an index of BPH progression, with a close correlation to the clinical parameters of BPH/BOO/LUTs [14–16]. A clinical trial showed that an *α*1-blocker, Tamsulosin, could significantly improve BPH/LUTs accompanied by an obvious decrease in the ultrasound-estimated bladder weight [16].

The involvement of several growth factors in the pathophysiology of an obstructed bladder has been identified [7, 17, 18]. BPH/BOO-induced changes of growth factors in bladder tissue regulate the remodeling of the bladder by various pathways and further impact bladder function. Nerve growth factor (NGF) plays a critical role in development neurobiology because of its important neuronal functions [19]. Neuronal hypertrophy has been observed in hypertrophic detrusors after partial BOO in rats, with an increased expression of NGF reported to be a major factor [20, 21]. In addition, a significant increase of NGF synthesis in inflammatory tissues has been described in patients and animal models [22], while the BOO-elicited inflammatory response has been well established in the urinary bladder [23, 24]. Multifunctional basic fibroblast growth factor (bFGF) is involved in the hypertrophic process of an obstructed bladder [25]. It has been reported that the level of bFGF gradually increases in rats with both mild and severe bladder -obstruction rats, and in vitro bFGF modulates both proliferation of and collagen deposition in bladder smooth muscle cells [25, 26]. Furthermore, bFGF may play a role in the bladder overactivity associated with BPH through modulating the gap junction intracellular communication among bladder smooth muscle cells [27]. Transformation growth factor-*β*1 (TGF-*β*1) has a critical role in regulating cell growth, morphogenesis, differentiation, and apoptosis. As a potent fibrogenic factor, TGF-*β*1 increases extracellular matrix accumulation by enhancing collagen synthesis and inhibiting protease production [28]. In addition, the blockage of TGF-*β*1-mediated signaling would decrease BOO-induced bladder hypertrophy, hyperplasia, and fibrosis [29, 30]. Furthermore, TGF-*β*1 plays a pivotal role in causing phenotypic transformation of bladder smooth muscle cells in rats with BOO [29]. Therefore, changes in TGF-*β*1 may be predictive in the evaluation of detrusor contractibility [26, 31]. In a BOO rat model, a negative correlation between detrusor contraction force and urine TGF-*β*1 was detected [31].

Qianlie Tongqiao Capsule (QTC), a patented Chinese drug, is composed of seven herbs, including* Astragalus mongholicus (Huang-qi)*,* Semen cuscutae (Tu-si-zi), Aulastomum gulo (Shui-zhi), Cinnamomum cassia (Rou-gui), Radix linderae (Wu-yao), Semen amomi amari (Yi-zhi-ren)*, and* Radix achyranthis bidentatae (Huai-niu-xi)*. BPH/LUTs with bladder dysfunction are considered to be “kidney deficiency and blood stasis” from the perspective of traditional Chinese medicine (TCM) [32–35]. QTC was identified to primarily tonify the kidney and promote blood circulation and water discharge. Clinically, QTC has significant therapeutic effects on patients with BPH and BPH-related bladder dysfunction [36, 37]. Previously, evidence-based results from a multicenter, randomized controlled, double-blind clinical trial conducted by Yunnan Provincial Hospital of Traditional Chinese Medicine (First Affiliated Hospital of Yunnan University of Traditional Chinese Medicine; Kunming, China), First Affiliated Hospital of Kunming Medical University (Kunming, China), and Kunming Municipal Hospital of Traditional Chinese Medicine (Third Affiliated Hospital of Yunnan University of Traditional Chinese Medicine; Kunming, China) showed that QTC significantly improved international prostate symptom scores (IPSS), quality of life (QoL) scores, and the maximum flow rate (Q_max_) and reduced residual volume in patients with BPH [36].

QTC has been clinically used to improve BPH-induced LUTs and bladder abnormalities. However, the functional mechanisms of QTC remain unknown. This study aims to investigate the effects of QTC on the bladder weigh, expression of NGF, bFGF, TGF-*β*1, and apoptosis-associated factors in the bladder tissue of rats with testosterone-induced BPH.

## 2. Materials and Methods

### 2.1. Animals and Grouping

A total of 40 adult male Sprague Dawley rats of a specific pathogen-free grade and weighing 220 ± 10 g (8 weeks old) purchased from the Laboratory Animal Center (Kunming Medical University, China) were used for this study. All animal experiments were performed in accordance with the Guide for the Care and Use of Laboratory Animals published by the National Institutes of Health. All animal protocols were approved by the Institutional Animal Care and Use Committee of Yunnan Provincial Hospital of Traditional Chinese Medicine (Kunming, China). All the rats were housed in a 12 h light/dark-cycle condition with appropriately controlled temperature (21 ± 2°C) and humidity (30%~70%) and acclimated for 1 week with free access to standard laboratory food and water* ad libitum*. All the rats were randomly divided into five groups: sham; model; and low, middle, and high dosage treatment with QTC, with eight animals in each group. Except for the sham group, the rats in the other groups were induced as the BPH model via castration with subcutaneous administration of testosterone propionate (TP; Tianjin Kingyork Group Co., Ltd., China).

### 2.2. Model Establishment

The rats in the model and QTC-treated groups underwent bilateral orchiectomy under aseptic conditions. The rats were anaesthetized with an intraperitoneal injection of 3% sodium pentobarbital (40 mg/kg; Sigma-Aldrich, St. Louis, MO). The rats were then fixed on the operating table, routinely disinfected, and their testicles were removed from the scrotum. The stumps and the incision were carefully ligated with 4-0 and 3-0 silk sutures, respectively. The sham operation was performed without bilateral orchiectomy. After a 1-week recovery period, BPH was induced in the model and drug-treated rat groups through daily subcutaneous injections of TP (5 mg/kg) dissolved in corn oil (Sigma-Aldrich, St. Louis, MO) for 4 weeks. Animals in the sham group received subcutaneous injections of only corn oil following the same schedule.

### 2.3. Preparation and Application of Drugs

**Figure 1:**
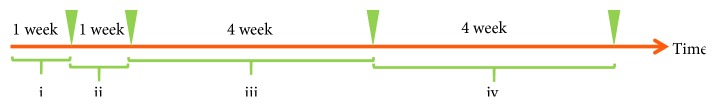
**Experimental protocol for model establishment and treatment processes**. (i) A time interval of 1 week for acclimation of the rats and a total of 40 rats were randomly divided into 5 groups: sham, model, low, middle, and high dosage of Qianlie Tongqiao Capsule (QTC), 8 rats in each group; (ii) a time interval of 1 week in which the sham group underwent sham surgery and the other groups received bilateral orchiectomy, and then all the rats took a postoperation recovery; (iii) a time interval of 4 weeks for inducement of BPH through daily subcutaneous injections of testosterone propionate (TP; 5 mg/kg) dissolved in corn oil (in the sham group, subcutaneous injections of only corn oil); (iv) a time interval of 4 weeks for QTC treatment on the groups along with daily subcutaneous injections of TP (5 mg/kg) dissolved in corn oil; the model group was orally force-fed with sterilized water along with daily subcutaneous injections of TP (5 mg/kg) dissolved in corn oil; the sham group was orally force-fed with sterilized water along with daily subcutaneous injections of corn oil. Finally, all the rats were euthanized.

QTC was formulated by Professor/Chief physician Chun-he Zhang, and produced and quality-controlled by the pharmaceutical preparation section of Yunnan Provincial Hospital of Traditional Chinese Medicine (Kunming, China). QTC (registered approval number [Z]20120001A) comprised seven Chinese herbs as represented in Table 1. The QTC powder appears brown, smells spicy, and tastes slightly bitter. The QTC powder was dissolved in water and treated with high-pressure sterilization to create solutions with a QTC concentration of 5% (5 g/100 mL), 10% (10 g/100 mL), and 20% (20 g/100 mL). Following the 4-week inducement of BPH, the rat drug-treatment groups received the different QTC dosages by gavage along with subcutaneous injections of TP (5 mg/kg) dissolved in corn oil for the next 4 weeks: low dosage, 0.38 g/kg/d with 5% QTC suspension; middle dosage, 0.76 g/kg/d with 10% QTC suspension; high dosage, 1.52 g/kg/d with 20% QTC suspension). The rats of the sham and model groups were orally force-fed with sterilized water along with subcutaneous injections of corn oil alone or corn oil plus TP (5 mg/kg), respectively (experimental protocol is shown in Figure 1).

### 2.4. Collection and Weighing of Bladder

After the final treatment, the rats fasted overnight and were euthanized group by group through an intraperitoneal injection of sodium pentobarbital (200 mg/kg). The bladders were immediately and carefully transected from the bladder neck and washed in 4°C sterile phosphate buffered saline (PBS) for the removal of residual blood and urine. After wiping away the residual liquid with aseptic gauze, each bladder was weighed and cut into two sections: one section was put into a freeze-safe storage tube and stored in liquid nitrogen, and the other section was fixed with 4% paraformaldehyde (Solarbio, China) for further assays.

### 2.5. Histological and Immunohistochemical (IHC) Staining

Excised bladder tissue samples were placed in 4% neutral buffered formalin for 48 h at room temperature and subsequently processed, embedded in paraffin, and sectioned at 4 *μ*m. The sections were stained with hematoxylin and eosin (H&E), according to the manufacturer's protocol (Beyotime Institute of Biotechnology, China), for histological evaluation. For the IHC staining, the sections were deparaffinized, rehydrated, and retrieved with heat-induced epitope retrieval. Endogenous peroxidase was inhibited with 3% hydrogen peroxide and nonspecific antigens were blocked with 5% bovine serum albumin (BSA; Sigma, USA). Immunostaining was carried out by incubating the tissue sections with NGF (1:250; Abcam, USA), bFGF (1:500; Santa Cruz Biotech, USA) and TGF-*β*1 (1:200; Abcam, USA) antibodies overnight at 4°C, rinsed with PBS, and incubated with a biotinylated secondary antibody (diluted 1:1000). IHC detection was performed with 3,3'-diaminobenzidine (DAB). The slides were photographed using a Nikon Eclipse 80i microscope (Nikon, Japan), and the images were captured from each slide at 200x magnification.

### 2.6. Western Blot Analysis

Total proteins of the rat bladders were determined using a BCA protein assay kit (Beyotime Institute of Biotechnology, China) following the manufacturer's protocol. Proteins were separated by sodium dodecyl sulfate-polyacrylamide gel electrophoresis and then transferred onto polyvinylidene difluoride membranes. After blocking with Tris buffer solution containing 5% nonfat milk for 1 h at 37°C, the membranes were incubated overnight at 4°C with primary antibodies against Bcl-2 (1:2000), Bax (1:1000), and *β*-actin (1:5000) (all antibodies from Abcam, USA). After thorough washing, the blots were incubated with horseradish peroxidase-conjugated secondary antibody (1:1000 in skim milk). The protein signals were visualized using QDYSSEY CLx Dual-Color Infrared Laser Imaging system (LI-COR Biosciences, USA).

### 2.7. Quantitative Reverse Transcription-Polymerase Chain Reaction (qRT-PCR) Assay

The frozen rat bladder detrusor samples were ground in a precooled mortar. Total RNA was extracted from the rat bladder detrusor samples using TRIzol reagent (TIANGEN Biotech Co., Ltd., China). Reverse transcription was performed using the PrimeScript™ RT reagent kit with gDNA eraser (TaKaRa, China) according to the manufacture's protocol. The specific primer sequences for RNA amplification were synthesized by Sangon Biotech (Shanghai, China) and are listed in Table 2. Real-Time PCR was performed using the ABI 7500 Real-Time PCR Instrument. The expression levels of the target genes relative to GAPDH were quantified following the 2^-ΔΔCt^ formula.

### 2.8. Statistical Analysis

Statistical analysis was performed by GraphPad Prism Version 6.00 for Windows (GraphPad Software, Inc., USA). All data were expressed as the mean ± standard error of the mean (SEM) and analyzed by one-way ANOVA with Tukey* post hoc* for multiple-group comparison. Results were considered statistically different when the* p* value was less than 0.05 and significantly different when the* p* value was less than 0.01.

## 3. Results

### 3.1. Effects of QTC on Bladder Weight and the Bladder Index in Rats with TP-Induced BPH

Outflow obstruction caused by BPH results in a durative increase in bladder weight and an obvious reduction in bladder contractility, which are signs of structural and functional changes in the obstructed bladder [38]. Following a 4-week BPH-inducement phase, different dosages of QTC were orally administered to the rats once daily for 4 weeks and the sham and model groups were deemed the negative and positive control, respectively. The body weight and bladder weight of all rats were recorded, and the bladder index was calculated as the bladder weight/body weight ratio. All of the results are listed in Table 3. The bladder weight and bladder index were significantly increased (*P* < 0.01) in the model group compared to the sham group. The QTC-treated groups showed different effects on bladder weight and the bladder index compared with the model group: low dosage of QTC (QTC Low) had no effect on either bladder weight or the bladder index; middle dosage of QTC (QTC Middle) had no effect on bladder weight but significantly decreased the bladder index (*P* < 0.01); high dosage of QTC (QTC High) had an obviously downregulated effect on both bladder weight (*P* = 0.039) and the bladder index (*P* < 0.01). There was no statistical difference in body weight among all the groups.

### 3.2. Effect of QTC on Histomorphology of the Bladder in Rats with TP-Induced BPH

**Figure 2:**
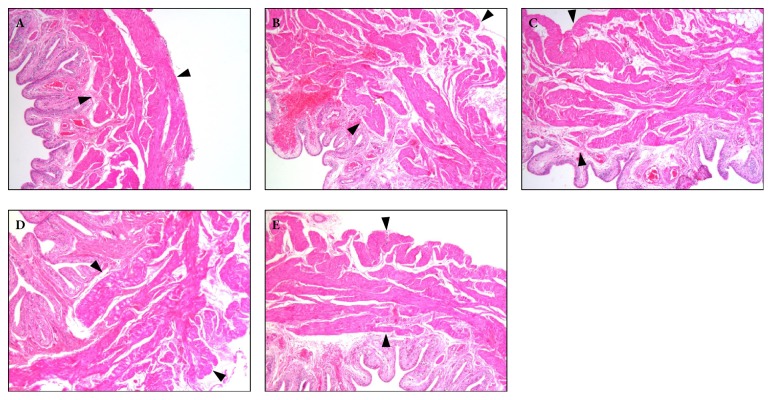
**Effect of QTC on histomorphology of the bladder in rats with TP-induced BPH**. (A) sham group; (B) model group; (C) low dosage QTC group; (D) middle-dosage QTC group; (E) high dosage QTC group. H&E staining was conducted, and representative results are shown in photomicrographs A ~ E (magnification × 50). The distance between the two black arrows in each photomicrograph denotes the thickness of the detrusor smooth muscle.

In the rats with TP-induced BPH, H&E staining revealed a remarkable increase of detrusor thickness. However, as shown in Figure 2, this phenomenon was clearly alleviated in the high dosage QTC group.

### 3.3. Effects of QTC on NGF Expression Level in the Bladder of Rats with TP-Induced BPH

**Figure 3:**
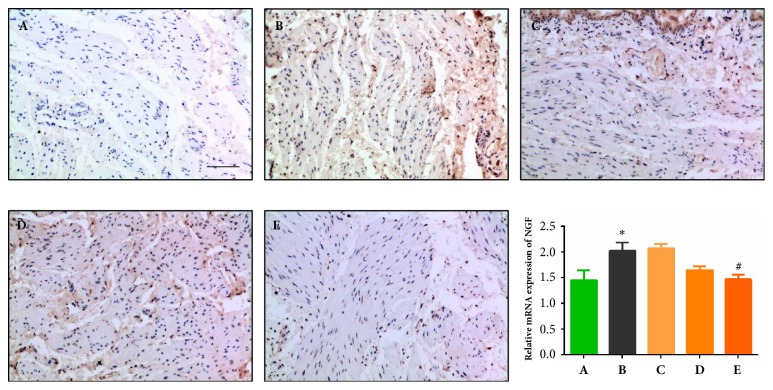
**Effect of QTC on NGF level in the bladder**. (A) Sham group; (B) model group; (C) low dosage QTC group; (D) middle-dosage QTC group; (E) high dosage QTC group. IHC analysis of NGF was conducted, and representative results are shown in photomicrographs (magnification × 200, scale bar = 100 *μ*m). Relative expressions of NGF mRNA are exhibited as bar graphs, compared to (A), ^*∗*^*P* < 0.05; compared to (B), ^#^*P *< 0.05.

Chronic BOO induced by BPH can stimulate NGF production, and this increase in NGF expression may be involved in bladder abnormalities and may positively correlate with the severity of overactive bladder [20, 39]. We collected bladder tissues at the end of this study for further IHC and qRT-PCR to detect changes in the NGF level among all the experimental groups. As shown in the micropictures of Figure 3, the stained dots represent NGF protein expression in bladder smooth muscle cells and are notably increased in the model group (B) compared to the sham group (A), while QTC treatment, especially a high dosage of QTC (E), alleviated the expression. The level of NGF mRNA was significantly elevated in the model group (B) compared to the sham group (A) (*P* < 0.05), and a high dosage of QTC markedly inhibited the expression (*P* = 0.032). However, the suppressing effect of the middle dosage of QTC on NGF mRNA expression was not statistically significant.

### 3.4. Effects of QTC on bFGF Expression Level in the Bladder of Rats with TP-Induced BPH

**Figure 4:**
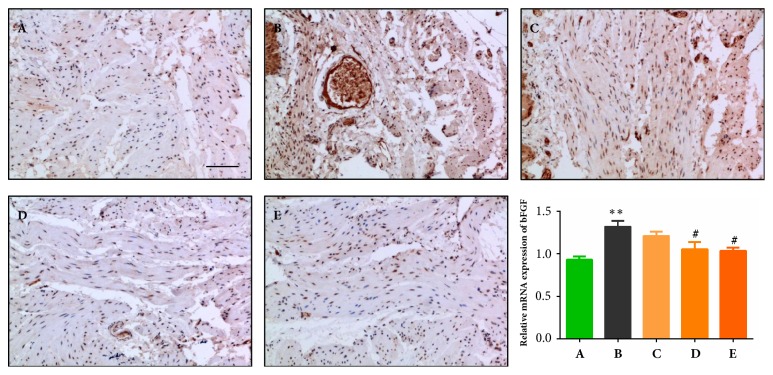
**Effect of QTC on bFGF level in the bladder**. (A) Sham group; (B) model group; (C) low dosage QTC group; (D) middle-dosage QTC group; (E) high dosage QTC group. IHC analysis of bFGF was conducted, and representative results are shown in photomicrographs (magnification × 200, scale bar = 100 *μ*m). Relative expressions of bFGF mRNA are exhibited as bar graphs, compared to (A), ^*∗∗*^*P *< 0.01; compared to (B), ^#^*P *< 0.05.

Previous studies demonstrated that bFGF has a regulatory effect on bladder smooth muscle remodeling with alterations of collagen deposition and the proliferation of bladder smooth muscle cells, and the bFGF level increased steadily throughout the time period of the obstruction, which may positively relate to the impairment of detrusor contractility [25, 40]. The bFGF levels among all groups in this study were detected by IHC and qRT-PCR. As shown in micropictures of Figure 4, the stained dots represent bFGF expression and are notably increased in the model group (B) compared to the sham group (A), with the expression suppressed by gradient-elevated QTC treatment, especially the middle- and high-dosages (D & E). Consistent with the IHC results, the expression of bFGF mRNA significantly increased in the model group (B) compared to the sham group (A) (*P* < 0.01), but both the middle- and high-dosages of QTC markedly inhibited it (*P* = 0.022,* P* = 0.012).

### 3.5. Effects of QTC on TGF-*β*1 Expression Level in the Bladder of Rats with TP-Induced BPH

**Figure 5:**
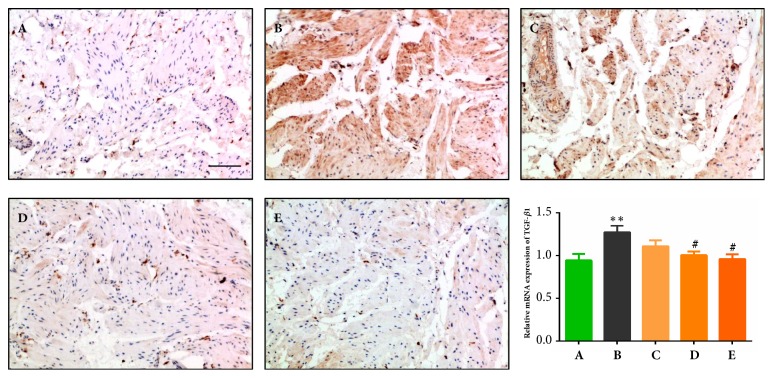
**Effect of QTC on TGF-**β**1 level in the bladder**. (A) Sham group; (B) model group; (C) low dosage QTC group; (D) middle-dosage QTC group; (E) high dosage QTC group. IHC analysis of TGF-*β*1 was conducted, and representative results are shown in photomicrographs (magnification × 200, scale bar = 100 *μ*m). Relative expressions of TGF-*β*1 mRNA are exhibited as bar graphs, compared to (A), ^*∗∗*^*P *< 0.01; compared to (B), ^#^*P *< 0.05.

It is known that TGF-*β*1 plays a key role in the progression of fibrosis, and the overexpression of TGF-*β*1 is involved in the pathophysiology of BPH/BOO-induced bladder dysfunction [30, 41]. In this study, IHC results (Figure 5) indicated that the stained range of TGF-*β*1 in the model group (B) obviously exceeded the sham group (A), while it was notedly reversed by the middle- and high-dosages of QTC (D & E). Also consistent with the IHC results, expression of TGF-*β*1 mRNA is upregulated significantly in the model group (B) compared to the sham group (A) (*P* < 0.01), but the middle- and high-dosages of QTC markedly inhibited it (*P* = 0.047,* P* = 0.015).

### 3.6. Effects of QTC on the mRNA and Protein Level of Bcl-2 and Bax and the Bcl-2/Bax Ratio in the Bladder of Rats with TP-Induced BPH

**Figure 6:**
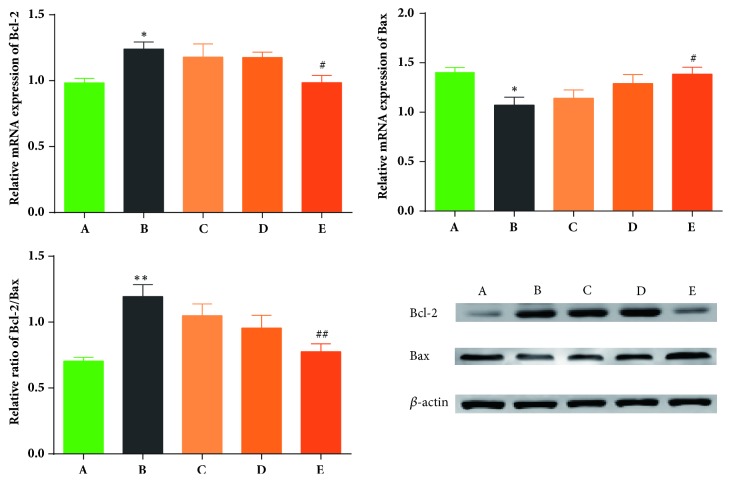
**Effects of QTC on the mRNA and protein level of Bcl-2 and Bax in the bladder. **(A) Sham group; (B) model group; (C) low dosage QTC group; (D) middle-dosage QTC group; (E) high dosage QTC group. Compared to (A), ^*∗*^*P *< 0.05 and ^*∗∗*^*P *< 0.01; compared to (B), ^#^*P *< 0.05 and ^##^*P *< 0.01.

The Bcl-2 gene family is closely involved in apoptosis pathways [42–44]. In our study, we investigated the expression of Bcl-2 and Bax mRNA. As shown in Figure 6, the levels of Bcl-2 mRNA and Bax mRNA are markedly upregulated and downregulated, respectively (*P* = 0.040,* P* = 0.028), in the model group (B) compared to the sham group (A). High-dosages of QTC (E) reversed the above-mentioned changes (compared with B,* P *< 0.05). In addition, the Bcl-2/Bax ratio was markedly elevated in the model group (B) compared with the sham group (A) (*P* < 0.01), but this elevation was significantly inhibited by high dosage QTC (E) (*P* < 0.01). In a similar manner, the changes in protein expression of Bcl-2 and Bax were consistent with the variety of gene expressions.

## 4. Discussion

Structural and functional abnormalities of the bladder are always secondary to the outlet obstruction caused by BPH [7, 45]. Thus, casting more light on the bladder problems of patients with BPH is important and necessary. In terms of drug treatment, *α*1-blockers, 5*α*-reductase inhibitors, and muscarinic receptor antagonists are clinically recommended to treat LUTs that are induced by benign prostatic obstruction [46, 47]. Nonetheless, while these drugs are effective, they may cause some adverse events [47]. Meanwhile, although patients with BPH can be asymptomatic [48] and impairment of the bladder can be gradual, bladder abnormalities suggestive of benign prostatic obstruction are always associated with the clinical progression of BPH. Thus, both treatment and prevention are critical for patients with BPH and LUTs.

TCM has established its own prevention-and-treatment system that benefits thousands of people in China and throughout the world [49]. TCM has advantages for the treatment of BPH and LUTs and protecting bladder function, and many scholars have carried out further studies to detect the potential mechanisms [50–57]. TCM classifies BPH and bladder abnormalities suggestive of BPH as “kidney deficiency and blood stasis”, and this viewpoint is a consensus among experts [33, 35]. QTC, a Chinese patented drug that we researched in the present study, is a representative Chinese formula consisting of seven herbs, which can tonify the kidney and promote blood circulation and water discharge. In our previous studies, QTC was validated as clinically efficacious and safe in patients with BPH-related LUTs and insufficient detrusor contractility [36, 37]. However, the functional mechanisms of QTC remain unclear. Indeed, the effective mechanisms of QTC should be complex and, in the present study, we primarily investigated the regulatory effect of QTC on bladder weight and several growth factors in the bladder of rats with TP-induced BPH.

An increase in bladder weight is associated with the progression of BPH and BOO [14–16], and this increase is the result of a hyperplastic bladder caused by prostatic obstruction [7]. Our results showed that bladder weight and the bladder index of rats with BPH were significantly higher than the sham group, and a high dosage of QTC had an obvious inhibitory effect. A middle dosage of QTC also markedly affected the bladder index, but its inhibitory effect on bladder weight had no statistical difference compared to the model group. Nevertheless, the present data shows that the dosage level of QTC influences bladder weight and the bladder index. Additionally, we can speculate that the effect of QTC on bladder weight may be associated with its inhibitory effect on abnormal bladder hyperplasia, since bladder hyperplasia is involved in the pathophysiology of BPH-induced changes of bladder structure. It is known that the Bcl-2 gene family is closely involved in apoptosis pathways [42–44]. Specifically, Bcl-2 impedes cell apoptosis but Bax promotes it. As shown in our results, Bcl-2 and Bax mRNA were notably upregulated and downregulated, respectively, in the bladder of rats with BPH, but a high dosage of QTC reversed these changes and simultaneously improved the Bcl-2/Bax ratio imbalance. In our opinion, apoptosis and the proliferation of normal bladder smooth muscle cells are under a dynamic balance that is dependent on the normal structure and function of the bladder. However, BPH and BOO can disrupt this balance and lead to bladder hypertrophy and hyperplasia, which are finally represented by an increased bladder weight, especially in the compensatory period. Thus, we speculate that the inhibitory effect of QTC on bladder weight is associated with its ability to recover the balance between Bcl-2 and Bax. However, the intervening mechanism needs to be investigated further.

In the present study, we also focused on several growth factors, such as NGF, bFGF, and TGF-*β*1 that are reported to be involved in the pathophysiological progression of bladder dysfunction induced by BPH [7]. Our results showed that protein and mRNA expression of NGF, bFGF, and TGF-*β*1 were elevated significantly in the bladder after prostatic obstruction, which was consistent with the other scholars' previous studies [21, 26, 29]. In addition, middle- and high-dosages of QTC inhibited the expression of these growth factors. However, the inhibitory effect of a middle dosage of QTC on the NGF level was not statistically significant. In fact, growth factors like NGF, bFGF, and TGF-*β*1 implement their physiological functions under normal conditions; however, overexpression of NGF, bFGF, and TGF-*β*1 in the bladder, which is induced by BPH and BOO, evolves into a pathophysiological condition. As the Chinese saying goes, “once a certain limit is reached, a change in the opposite direction is inevitable”. Our results indicate that the clinical effect of QTC on patients with BPH-caused LUTs and bladder dysfunction may be associated with the regulation of these growth factors by QTC to a modest level, but the specific pharmacological mechanism needs to be clarified in a further study. Moreover, animal experiments cannot be strictly representative of what happens in the human bladder during prostatic obstruction, which is a limitation of this study.

## 5. Conclusions

We found that QTC, especially taken at a high dosage, had a significant inhibitory effect on bladder weight and overexpression of NGF, bFGF, and TGF-*β*1 in the bladder of rats with BPH. In addition, QTC regulates mRNA and protein expression of Bcl-2 and Bax in the bladder after prostatic obstruction. These results may reveal functional mechanisms of QTC, but further studies are still needed.

## Figures and Tables

**Table 1:** Herbal ingredients of Qianlie Tongqiao Capsule (QTC).

**Chinese name**	**Full scientific name**	**Part used**	**Proportion**
Huang-Qi (黃芪)	*Astragalus mongholicus*	Dried root	21.6%
Shui-Zhi (水蛭)	*Aulastomum gulo*	Dried body	4.3%
Tu-Si-Zi (*菟絲*子)	*Semen cuscutae*	Dried seed	14.4%
Rou-Gui (*肉桂*)	*Cinnamomum cassia*	Dried bark	21.6%
Wu-Yao (*烏藥*)	*Radix linderae*	Dried rhizome	21.6%
Yi-Zhi-Ren (*益智仁*)	*Semen amomi amari*	Dried fruit	5.7%
Huai-Niu-Xi (*懷牛膝*)	*Radix achyranthis bidentatae*	Dried root	10.8%

**Table 2:** Primers for different target genes used in this study.

**Gene**	**Primers**	**Product size (bp)**
NGF	Sense:ACAGATAGCAATGTCCCAGAGG	243
	Anti-sense:ATCCAGAGTGTCCGAAGAGGTG	

bFGF	Sense:GAAGAGCGACCCACACGTCAAAC	67
	Anti-sense:TCCCTTGATGGACACAACTCCTCTC	

TGF-*β*1	Sense:GAGAGCCCTGGATACCAACTACTGC	93
	Anti-sense:CAACCCAGGTCCTTCCTAAAGTCAA	

Bax	Sense:GGTGGTTGCCCTTTTCTACTTTGC	113
	Anti-sense:GGTGGTTGCCCTTTTCTACTTTGC	

Bcl-2	Sense:GGGCTACGAGTGGGATACTGGAG	101
	Anti-sense:CGGGCGTTCGGTTGCTCT	

GAPDH	Sense:CCTTCCGTGTTCCTACCCC	131
	Anti-sense:GCCCAGGATGCCCTTTAGTG	

**Table 3:** Effects of QTC on bladder weight and bladder index in TP-induced BPH rats.

Groups	Body weight (g)	Bladder weight (mg)	Bladder index (mg/g)
Sham	403.7 ± 10.8	135.08 ± 7.04	0.333 ± 0.011
Model	393.7 ± 7.6	198.77 ± 4.04^*∗∗*^	0.505 ± 0.007^*∗∗*^
QTC Low	409.7 ± 10.0	204.32 ± 6.53	0.498 ± 0.006
QTC Middle	402.7 ± 9.0	183.17 ± 6.45	0.454 ± 0.006^##^
QTC High	412.8 ± 9.9	173.15 ± 5.83^#^	0.419 ± 0.009^##^

All data were presented as mean ± SEM, 8 rats in each group; bladder index was calculated as the bladder weight/body weight ratio. Compared to the sham group, ^*∗∗*^*P *< 0.01, compared to the model group, ^#^*P* < 0.05, and ^##^*P* < 0.01. QTC Low: low dosage of Qianlie Tongqiao Capsule, QTC Middle: middle dosage of Qianlie Tongqiao Capsule, and QTC High: high dosage of Qianlie Tongqiao Capsule.

## Data Availability

The data used to support the findings of this study are available from the corresponding author upon request.
